# Sodium oligomannate targeting of the cGAS‐STING‐IFN‐I pathway for Alzheimer's disease

**DOI:** 10.1002/alz70855_102610

**Published:** 2025-12-23

**Authors:** Qiong Wang, Duntao Huang, Linbin Dai, Feng Gao, Yong Shen

**Affiliations:** ^1^ Department of Neurology, Institute on Aging and Brain Disorders, The First Affiliated Hospital of USTC, Division of Life Sciences and Medicine, University of Science and Technology of China, Hefei, Anhui, China; ^2^ Neurodegenerative Disorder Research Center, Division of Life Sciences and Medicine, University of Science and Technology of China, Hefei, Anhui, China

## Abstract

**Background:**

Recent studies demonstrated that therapeutic effects of sodium oligomannate on cognitive function in both mouse models and patients with Alzheimer's disease (AD). However, the mechanism remains inconclusive.

**Method:**

Here, we employed 5xFAD and APP^NL‐G‐F^ AD mouse models treating with sodium oligomannate or vehicle for 8 or 16 weeks. Then, several behavior tests and AD pathology were exanimated. Brain bulk RNA‐seq was performed to explore the molecular mechanisms. Simultaneously, we observed changes in blood indicators among AD patients taking with sodium oligomannate for 9 months.

**Result:**

We found that sodium oligomannate treatment rescued spatial and learning memory, reduced pro‐inflammatory activation of microglia, decreased dense‐core amyloid plaques and the levels of plasma Aβ42, lowered levels of phosphorylated tau proteins, and less neuronal synapse damage both in female and male AD mouse models. Through bulk RNA sequencing, we revealed that sodium oligomannate reversed the abnormal activation of neuroinflammation and type I interferon signaling pathway, and up‐regulated DNA repair related genes in the brain. Furthermore, our study discovered that sodium oligomannate alleviated DNA damage and inhibited the activation of the cGAS‐STING‐type I interferon pathway in AD mouse model. Notably, we found sodium oligomannate reduced the Aβ42/ Aβ40 ratio as well as pro‐inflammatory markers in the peripheral blood of AD patients, consistent with the results from our basic research.

**Conclusion:**

These findings highlight the mechanism of sodium oligomannate on Alzheimer's disease.

